# Euphrasia and Cepa

**Published:** 1887-07

**Authors:** 


### EUPHRASIA AND CEPA.

Running coryza, increasing in the evening, with flow of tears and a cough, indicates *Euphrasia.* Especially if the discharge from the nose is mild, the tears sharp, and the cough worse during the day.

If the discharge from the nose is acrid, the tears mild, and the cough increases in the evening, sometimes with a pain as if the larynx would be torn, *Cepa* is indicated. Colds after damp northeastern winds mostly correspond to *Cepa. Euphrasia* has complaints from very windy weather.

*Euphrasia* predominantly affects the provers first on the right side and afterward on the left, but generally more on the right side. In most all the cures reported, the disease extended from left to right. In *Cepa* it seems to be the rule that the symptoms, with the prover, go in the upper half of the body, from right to left, and in the lower half from left to right. In most of the colds where *Cepa* had a decided curative influence, the disease began on the left side and extended afterward to the right. If also the opposite with diseases of the lower half of the body, is yet to be ascertained.

